# *Apophysomycesthailandensis* (Mucorales, Mucoromycota), a new species isolated from soil in northern Thailand and its solubilization of non-soluble minerals

**DOI:** 10.3897/mycokeys.45.30813

**Published:** 2019-01-29

**Authors:** Surapong Khuna, Nakarin Suwannarach, Jaturong Kumla, Jomkhwan Meerak, Wipornpan Nuangmek, Tanongkiat Kiatsiriroat, Saisamorn Lumyong

**Affiliations:** 1 Department of Biology, Faculty of Science, Chiang Mai University, Chiang Mai 50200, Thailand Chiang Mai University Chiang Mai Thailand; 2 PhD Degree Program in Applied Microbiology, Department of Biology, Faculty of Science, Chiang Mai University, Chiang Mai 50200, Thailand University of Phayao Phayao Thailand; 3 Center of Excellence in Microbial Diversity and Sustainable Utilization, Chiang Mai University, Chiang Mai 50200, Thailand Academy of Science, The Royal Society of Thailand Bangkok Thailand; 4 Faculty of Agriculture and Natural Resources, University of Phayao, Phayao 56000, Thailand Chiang Mai University Chiang Mai Thailand; 5 Center of Excellence for Renewable Energy, Chiang Mai University, Chiang Mai 50200, Thailand University of Phayao Phayao Thailand; 6 Academy of Science, The Royal Society of Thailand, Bangkok 10300, Thailand Academy of Science, The Royal Society of Thailand Bangkok Thailand

**Keywords:** *
Apophysomyces
*, mineral solubilization, soil fungi, taxonomy

## Abstract

A new species of soil fungi, described herein as *Apophysomycesthailandensis*, was isolated from soil in Chiang Mai Province, Thailand. Morphologically, this species was distinguished from previously described *Apophysomyces* species by its narrower trapezoidal sporangiospores. A physiological determination showed that *A.thailandensis* differs from other *Apophysomyces* species by its assimilation of D-turanose, D-tagatose, D-fucose, L-fucose, and nitrite. A phylogenetic analysis, performed using combined internal transcribed spacers (ITS), the large subunit (LSU) of ribosomal DNA (rDNA) regions, and a part of the histone 3 (H3) gene, lends support to our the finding that *A.thailandensis* is distinct from other *Apophysomyces* species. The genetic distance analysis of the ITS sequence supports *A.thailandensis* as a new fungal species. A full description, illustrations, phylogenetic tree, and taxonomic key to the new species are provided. Its metal minerals solubilization ability is reported.

## Introduction

The genus *Apophysomyces*, proposed by Misra et al. (1979) with *A.elegans* as type species, belongs to the family Saksenaeaceae of the order Mucorales (Hoffmann et al. 2013). This genus is mainly characterized by pyriform sporangia, conspicuous funnel- and/or bell-shaped apophyses, and subhyaline, smooth-walled sporangiospores (Misra et al. 1979; Cooter et al. 1990; Alvarez et al. 2010). *Apophysomyces* is commonly found in soil, decaying vegetation, and detritus, and it has been reported to cause severe human infections in temperate and tropical regions (Misra et al. 1979; Cooter et al. 1990; Chakrabarti et al. 2003; Alvarez et al. 2010; Bonifaz et al. 2014). Currently, there are five known *Apophysomyces* species including *A.elegans* P.C. Misra, K.J. Srivast. & Lata (Misra et al. 1979), *A.ossiformis* E. Álvarez, Stchigel, Cano, Deanna A. Sutton & Guarro (Alvarez et al. 2010), *A.trapeziformis* E. Álvarez, Stchigel, Cano, Deanna A. Sutton & Guarro (Alvarez et al. 2010), *A.variabilis* E. Álvarez, Stchigel, Cano, Deanna A. Sutton & Guarro (Alvarez et al. 2010), and *A.mexicanus* A. Bonifaz, Cano, Stchigel & Guarro (Bonifaz et al. 2014).

During the isolation of non-soluble mineral solubilizing fungi from agricultural soil in northern Thailand, we found a particular population of *Apophysomyces* which we describe here as a new species based on morphological, molecular, and physiological characteristics. To confirm its taxonomic status, the phylogenetic relationship was determined by analysis of the combined sequence dataset of the ITS and LSU of rDNA, and part of the histone 3 gene.

## Materials and methods

### Fungal isolation

Soil samples were collected from agricultural areas of Mae Wang District, Chiang Mai Province, Thailand. The samples were air-dried at room temperature for 3 d, sieved and mixed through a 2 mm mesh prior to isolation of fungi by serial dilution. The dilution spread plate method was used with three serial dilutions in 0.5% NaCl solution. After dilution, 0.1 ml of suspension was spread on modified Aleksandrov agar (5.0 g glucose, 0.5 g MgSO_4_•7H_2_O, 0.1 g CaCO_3_, 0.005 g FeCl_3_, 2.0 g Ca_3_PO_4_, 3.0 g K_2_HPO_4_, and 15.0 g agar, pH 7.0, in 1 L of deionized water) for detection of non-soluble mineral solubilizing fungi. The plates were incubated at 30 °C in darkness for 5 d. Colonies which produced clear zones were considered mineral solubilizing strains and were selected for further studies.

### Morphological studies and growth observation

The colonies’ morphology on potato dextrose agar (PDA; CONDA, Spain), Czapek agar (CZA; Difco, France), and malt extract agar (MEA; Difco, France) was observed after 5 d of incubation in darkness at 37 °C. Three replicates were made in each medium. The colony diameter was measured. Micromorphological features were examined using a light microscope (Olympus CX51, Japan) following the methods described by Alvarez et al. (2010). The anatomical features were from at least 50 measurements of each structure.

### Physiological studies

Carbon source assimilation profiles were determined with the API 50CH commercial kit (bioMérieux, France), following the methods described by Schwarz et al. (2007). To obtain sufficient sporulation, all isolates were cultured for 1 week on CZA at 37 °C. A final concentration of 5 × 10^5^ spores/ml was prepared in 20 ml of yeast nitrogen base containing 0.5 g/l of chloramphenicol and 0.1% Bacto agar, and each well of the strips was inoculated with 300 µl of the spore containing medium. The inoculated API 50CH strips were incubated for 48–72 h at 37 °C in darkness. After incubation, the strips were read visually and growth or lack of growth was noted. Weak growth was considered as a positive result.

For nitrogen source assimilation we prepared inoculum as described above, but the yeast nitrogen base broth was replaced by carbon nitrogen base broth, and testing was performed in sterile, disposable, multiwell microplates. The medium with the nitrogen sources was dispensed into the wells in 150 µl, and each well was inoculated with 50 µl of the spore containing medium. The microplates were incubated at 37 °C in darkness for 48–72 h. Growth on NaCl (2%, 5%, 7%, and 10%), 2% MgCl_2_ and 0.1% cycloheximide was determined. All tests were performed in three replicates.

### Molecular studies

Genomic DNA of five day-old fungal mycelia on CZA was extracted using the fungal Genomic DNA Extraction Mini Kit (FAVOGEN, Taiwan). The ITS region of DNA was amplified by polymerase chain reactions (PCR) using ITS4 and ITS5 primers (White et al. 1990), the LSU of rDNA gene were amplified with NL1 and NL4 primers (Kurtzman and Robnett 1998), and histone 3 (H3) gene was amplified with the H3-1a and H3-1b primers (Glass and Donaldson 1995). The amplification program for these three domains were performed in separated PCR reaction and consisted of an initial denaturation at 95 °C for 5 min, followed by 35 cycles of denaturation at 95 °C for 30 s, annealing at 52 °C for 30 s (ITS); 52 °C for 45 s (LSU), and 54 °C for 1 min (H3), and extension at 72 °C for 1 min. Negative controls lacking fungal DNA were run for each experiment to check for any contamination of the reagents. PCR products were checked on 1% agarose gels stained with ethidium bromide under UV light and purified using NucleoSpin Gel and PCR Clean-up Kit (Macherey-Nagel, Germany). The purified PCR products were directly sequenced. Sequencing reactions were performed and sequences were automatically determined in a genetic analyzer at 1^st^ Base Company (Kembangan, Malaysia) using the same PCR primers mentioned above. Sequences were used to query GenBank via BLAST (http://blast.ncbi.nlm.nih.gov).

Details of the sequences used for phylogenetic analysis obtained from this study and from previous studies are provided in Table 1. The multiple sequence alignment was carried out using MUSCLE (Edgar 2004), and a combined ITS, LSU, and H3 alignments were deposited in TreeBASE under the study ID 23168. The combined ITS, LSU and H3 sequences dataset consisted of 28 taxa and the aligned dataset comprised 1991 characters including gaps (ITS: 1–942, LSU: 943–1620, and H3: 1621–1991). A maximum likelihood (ML) phylogenetic tree was constructed using RAxML v. 7.0.3 (Stamatakis 2006), applying the rapid bootstrapping algorithm for 1000 replications. *Saksenaeavasiformis* ATCC 60625 and *S.erythrospora* UTHSC 08-3606 were used as the outgroup. The ML trees were plotted with TreeView32 (Page 2001). Clades with bootstrap values (BS) ≥ 70% were considered as significantly supported (Hillis and Bull 1993). The best-fit substitution model for Bayesian inference algorithm was estimated by jModeltest v. 2.1.10 (Darriba et al. 2012) using Akaike information criterion. Bayesian phylogenetic analyses were carried out using the Metropolis-coupled Markov chain Monte Carlo (MCMCMC) method in MrBayes v. 3.2 (Ronquist et al. 2012), under a GRT+I+G model. Markov chains were run for one million generations, with six chains and random starting trees. The chains were sampled every 100 generations. Among these, the first 2000 trees were discarded as the burn-in phase of each analysis and the resulting trees were used to calculate Bayesian posterior probabilities. Bayesian posterior probabilities (PP) ≥ 0.95 were considered as a significant support (Alfaro et al. 2003). Pairwise genetic distances (proportions of variable sites) within and between five *Apophysomyces* species were computed using MEGA v. 6 (Tamura et al. 2013), with pairwise deletion of gaps and missing data.

**Table 1.** Sequences used for phylogenetic analysis. Type species of *Apophysomyces* are in bold.

Taxa	Strain/isolate	GenBank accession number			References
		ITS	D1/D2 domain	H3	
*** Apophysomyces elegans ***	CBS 476.78	FN556440	FN554249	FN555155	Alvarez et al. 2010
* Apophysomyces elegans *	CBS 477.78	FN556437	FN554250	FN555154	Alvarez et al. 2010
* Apophysomyces elegans *	FMR 12015	HE664070	–	–	Da Cunha et al. 2012
*** Apophysomyces variabilis ***	CBS 658.93	FN556436	FN554258	FN555161	Alvarez et al. 2010
* Apophysomyces variabilis *	UTHSC 06-4222	FN556428	FN554255	FN555162	Alvarez et al. 2010
* Apophysomyces variabilis *	UTHSC 03-3644	FN556431	FN554259	FN555158	Alvarez et al. 2010
* Apophysomyces variabilis *	GMCH 480/07	FN556442	FN554253	FN555163	Alvarez et al. 2010
* Apophysomyces variabilis *	IMI 338332	FN556438	FN554257	FN555159	Alvarez et al. 2010
* Apophysomyces variabilis *	IMI 338333	FN556439	FN554256	FN555160	Alvarez et al. 2010
* Apophysomyces variabilis *	GMCH 211/09	FN556443	FN554254	FN555164	Alvarez et al. 2010
* Apophysomyces variabilis *	FMR 13881	LT837923	LT837927	–	Unpublished
* Apophysomyces variabilis *	FMR 13217	LT837922	LT837926	–	Unpublished
* Apophysomyces variabilis *	FMR 12016	HE664071	–	–	Da Cunha et al. 2012
* Apophysomyces variabilis *	GMCH M333/05	FN813491	–	–	Guarro et al. 2011
* Apophysomyces variabilis *	GMCH M52/05	FN813490	–	–	Guarro et al. 2011
*** Apophysomyces trapeziformis ***	UTHSC 08-1425	FN556429	FN554261	FN555168	Alvarez et al. 2010
* Apophysomyces trapeziformis *	UTHSC 08-2146	FN556430	FN554260	FN555169	Alvarez et al. 2010
* Apophysomyces trapeziformis *	UTHSC 06-2356	FN556427	FN554262	FN555167	Alvarez et al. 2010
* Apophysomyces trapeziformis *	UTHSC 04-891	FN556433	FN554264	FN555165	Alvarez et al. 2010
* Apophysomyces trapeziformis *	UTHSC R-3841	FN556434	FN554263	FN555166	Alvarez et al. 2010
*** Apophysomyces ossiformis ***	UTHSC 04-838	FN556432	FN554252	FN555157	Alvarez et al. 2010
* Apophysomyces ossiformis *	UTHSC 07-204	FN556435	FN554251	FN555156	Alvarez et al. 2010
*** Apophysomyces mexicanus ***	CBS 136361	HG974255	HG974256	HG974254	Bonifaz et al. 2014
* Apophysomyces thailandensis *	SDBR-CMUS24	MH733250	MH733253	MH733256	This study
*** Apophysomyces thailandensis ***	SDBR-CMUS26	MH733251	MH733254	MH733257	This study
* Apophysomyces thailandensis *	SDBR-CMUS219	MH733252	MH733255	MH733258	This study
* Saksenaea vasiformis *	ATCC 60625	FR687323	HM776675	–	Alvarez et al. 2010
* Saksenaea erythrospora *	UTHSC 08-3606	FR687328	HM776680	–	Alvarez et al. 2010

### The non-soluble minerals solubilization ability

This experiment was carried out using basal medium (10.0 g glucose, 0.5 g (NH)_4_SO_4_, 0.2 g NaCl, 0.1 g MgSO_4_•7H_2_O, 0.2 g KCl, 0.5 g yeast extract, 0.002g MnSO_4_•H_2_O, and 15.0 g agar per liter of deionized water, pH 7.0) with addition of non-soluble metal minerals including Ca_3_(PO_4_)_2_, CaCO_3_, CuCO_3_•Cu(OH)_2_, CuO, CoCO_3_, FePO_4_, MgCO_3_, MnO, ZnCO_3_, ZnO, feldspar (KAlSi_3_O_8_), and kaolin (Al_2_Si_2_O_5_(OH)_4_) to the desired final concentration of 0.5% according to the method described by Fomina et al. (2005). The medium was autoclaved at 121 °C for 15 min. After autoclaving, for each experiment, 25 ml of test media was poured into Petri dishes. Mycelial inocula were prepared by growing the fungus on CZA at 30 °C in darkness for 7 d. Mycelial plugs (5 mm in diameter) from the periphery of the growing colony were then used to inoculate the center of the tested media. All plates were incubated at 30 °C in darkness for 4 d. Colony diameter and solubilization zone (halo zone) were measured. Solubilization index (SI) was calculated as the halo zone diameter divided by the fungal colony diameter (Vitorino et al. 2012, Kumla et al. 2014). SI values of less than 1.0, between 1.0 and 2.0, and more than 2.0 were regarded as low, medium, and high solubilization activities, respectively. Three replications were made in each treatment.

### Statistical analysis

The data were analyzed by one-way analysis of variance (ANOVA) by SPSS program version 16.0 (SPSS Inc., USA) for Windows, and Tukey’s range test was used for significant differences (*P* <0.05) between treatments.

## Results

### Growth observation and physiological studies

Mycelial growth of the three *A.thailandensis* isolates on three different agar media and at different temperatures is presented in Table 2. PDA promoted the best mycelial growth followed by CZA, and MEA. All isolates grew at temperatures ranging from 20–42 °C. The highest growth rate was observed on PDA at 37 °C.

**Table 2.** Growth rate of *Apophysomycesthailandensis* on different media and at different temperatures.

Medium	Temperature (°C)	Isolate/growth rate (mm/day)		
		SDBR-CMUS24	SDBR-CMUS26 (Holotype)	SDBR-CMUS219
PDA	4	–	–	–
	20	5.78 ± 0.51 i	5.78 ± 0.19 jk	5.67 ± 0.67 i
	25	8.58 ± 0.76 g	8.67 ± 0.76 g	8.83 ± 0.88 f
	30	28.33 ± 0.00 b	28.33 ± 0.00 b	28.33 ± 0.00 b
	37	40.64 ± 0.00 a	45.04 ± 0.00 a	42.64 ± 0.00 a
	42	16.73 ± 0.47 d	17.00 ± 0.00 d	16.89 ± 0.19 d
	45	–	–	–
	50	–	–	–
MEA	4	–	–	–
	20	3.64 ± 0.62 k	3.55 ± 0.16 l	3.69 ± 0.36 k
	25	5.89 ± 019 i	6.11 ± 0.19 ij	6.00 ± 0.33 hi
	30	7.00 ± 0.71 h	7.57 ± 0.74 h	6.95 ± 0.70 gh
	37	9.80 ± 1.00 f	9.93 ± 1.10 f	9.07 ± 0.99 f
	42	6.13 ± 0.63 i	6.38 ± 0.57 i	6.08 ± 0.62 hi
	45	–	–	–
	50	–	–	–
CZA	4	–	–	–
	20	4.60 ± 0.20 j	4.93 ± 0.76 k	4.67 ± 0.99 j
	25	7.89 ± 0.35 g	8.33 ± 0.76 gh	7.28 ± 0.19 g
	30	17.00 ± 0.00 d	17.00 ± 0.00 d	17.00 ± 0.00 d
	37	21.25 ± 0.00 c	21.25 ± 0.00 c	21.25 ± 0.00 c
	42	13.79 ± 0.46 e	14.09 ± 0.13 e	13.94 ± 0.39 e
	45	–	–	–
	50	–	–	–

PDA = potato dextrose agar, MEA = malt extract agar and CZA = Czapek agar. “-” = no growth. Value with the different letters with in the same column indicated the significant difference at *P* <0.05 according to Tukey’s range test

Carbon assimilation profiles of the three strains of *A.thailandensis* are shown in Table 3. Assimilation patterns of all strains were positive for 23 carbon sources (amidon, D-adonitol, D-arabitol, D-fructose, D-fucose, D-glucose, D-lyxose, D-maltose, D-mannitol, D-mannose, D-melezitose, D-ribose, D-sorbitol, D-tagatose, D-trehalose, D-turanose, D-xylose, glycerol, glycogen, L-arabinose, L-fucose, *N*-acetyl-glucosamine and xylitol). Variability in nitrogen assimilation and tolerance to NaCl, MgCl_2_, and cycloheximide of the three strains of *A.thailandensis* are presented in Table 4. All strains were positive for 10 nitrogen sources (arginine, creatine, L-cysteine, L-leucine, L-lysine, L-ornithine, L-proline, L-tryptophan, nitrate and nitrite). All strains were able to grow on 2% MgCl_2_, but could not grow on 2%, 5%, 7%, and 10% NaCl, and on 0.1% cycloheximide.

**Table 3.** Carbon assimilation profiles for *Apophysomyces* species obtained with API 50 CH strips.

Carbon source	* A. thailandensis * ^a^			* A. elegans * ^b^	* A. mexicanus * ^c^	* A. ossiformis * ^b^	* A. trapeziformis * ^b^	* A. variabilis * ^b^
	SDBR-CMUS24	SDBR-CMUS26^T^	SDBR-CMUS219	CBS 476.78 ^T^	CBS 136361 ^T^	UTHSC 04-838 ^T^	UTHSC 08-1425 ^T^	CBS 658.93 ^T^
GLY (glycerol)	+	+	+	+	+	+	+	+
ERY (erythritol)	–	–	–	–	–	–	–	–
DARA (D-arabinose)	–	–	–	–	–	–	–	–
LARA (L-arabinose)	+	+	+	+	–	+	+	+
RIB (D-ribose)	+	+	+	+	+	+	+	+
DXYL (D-xylose)	+	+	+	+	+	+	+	+
LXYL (L-xylose)	–	–	–	–	–	–	–	–
ADO (D-adonitol)	+	+	+	+	+	+	+	+
MDX (*methyl*-ß-D-xylopyranoside)	–	–	–	–	–	–	–	–
GAL (D-galactose)	–	–	–	–	–	–	–	–
GLU (D-glucose)	+	+	+	+	+	+	+	+
FRU (D-fructose)	+	+	+	+	+	+	+	+
MNE (D-mannose)	+	+	+	+	+	+	+	+
SBE (L-sorbose)	–	–	–	–	–	–	–	–
RHA (L-rhamnose)	–	–	–	–	–	–	–	–
DUL (dulcitol)	–	–	–	–	–	–	–	–
INO (inositol)	–	–	–	–	–	–	–	–
MAN (D-mannitol)	+	+	+	+	+	+	+	+
SOR (D-sorbitol)	+	+	+	+	+	+	+	+
MDM (*methyl*-D-mannopyranoside)	–	–	–	–	–	–	–	–
MDG (*methyl*-D-glucopyranoside)	–	–	–	–	–	–	–	–
NAG (*N*-acetyl-glucosamine)	+	+	+	+	+	+	+	+
AMY (amygdalin)	–	–	–	–	–	–	–	–
ARB (arbutin)	–	–	–	–	–	–	–	–
ESC (esculin)	–	–	–	+	–	–	–	–
SAL (salicin)	–	–	–	–	–	–	–	–
CEL (D-cellobiose)	–	–	–	+	–	+	+	+
MAL (D-maltose)	+	+	+	+	+	+	+	+
LAC (D-lactose)	–	–	–	–	–	–	–	–
MEL (D-melibiose)	–	–	–	–	–	–	–	–
SAC (D-saccharose)	–	–	–	–	–	–	–	–
TRE (D-trehalose)	+	+	+	+	+	+	+	+
INU (inulin)	–	–	–	–	–	–	–	–
MLZ (D-melezitose)	+	+	+	+	–	+	+	+
RAF (D-raffinose)	–	–	–	–	–	–	–	–
AMD (amidon)	+	+	+	+	–	+	+	+
GLYG (glycogen)	+	+	+	+	+	+	+	+
XLT (xylitol)	+	+	+	+	+	+	+	+
GEN (gentiobiose)	–	–	–	–	–	–	–	–
TUR (D-turanose)	+	+	+	–	–	–	–	–
LYX (D-lyxose)	+	+	+	–	+	+	–	–
TAG (D-tagatose)	+	+	+	–	–	–	–	–
DFUC (D-fucose)	+	+	+	–	–	–	–	–
LFUC (L-fucose)	+	+	+	–	–	–	–	–
DARL (D-arabitol)	+	+	+	+	+	+	+	+
LARL (L-arabitol)	–	–	–	+	+	+	+	+
GNT (potassium gluconate)	–	–	–	–	+	–	–	–
2KG (potassium 2-keto- gluconate)	–	–	–	–	–	–	–	–
5KG (potassium 5-keto- gluconate)	–	–	–	–	–	–	–	–

^a^This study, ^b^Alvarez et al. (2010) and ^c^Bonifaz et al. (2014)

**Table 4.** Nitrogen assimilation and tolerance to chemical compounds for *Apophysomyces* species.

Nitrogen source and other tests	* A. thailandensis * ^a^			* A. elegans * ^b^	* A. mexicanus * ^c^	* A. ossiformis * ^b^	* A. trapeziformis * ^b^	* A. variabilis * ^b^
	SDBR-CMUS24	SDBR-CMUS26 ^T^	SDBR-CMUS219	CBS 476.78 ^T^	CBS 136361 ^T^	UTHSC 04-838 ^T^	UTHSC 08-1425 ^T^	CBS 658.93 ^T^
Creatine	+	+	+	+	+	+	+	+
L-lysine	+	+	+	+	+	+	+	+
Nitrate	+	+	+	+	+	+	+	+
Nitrite	+	+	+	–	–	–	–	–
L-tryptophan	+	+	+	+	+	+	+	+
L-proline	+	+	+	+	+	+	+	+
L-leucine	+	+	+	+	+	+	+	+
L-ornithine	+	+	+	+	+	+	+	+
L-cysteine	+	+	+	+	+	+	+	+
Arginine	+	+	+	+	+	+	+	+
2% NaCl	–	–	–	+	+	+	+	+
5% NaCl	–	–	–	–	–	–	–	–
7% NaCl	–	–	–	–	–	–	–	–
10% NaCl	–	–	–	–	–	–	–	–
2% MgCl_2_	+	+	+	+	+	+	+	+
Cycloheximide 0.1%	–	–	–	–	–	–	–	–

^a^This study, ^b^Alvarez et al. (2010) and ^c^Bonifaz et al. (2014)

### Phylogenetic results

**Figure 1.**
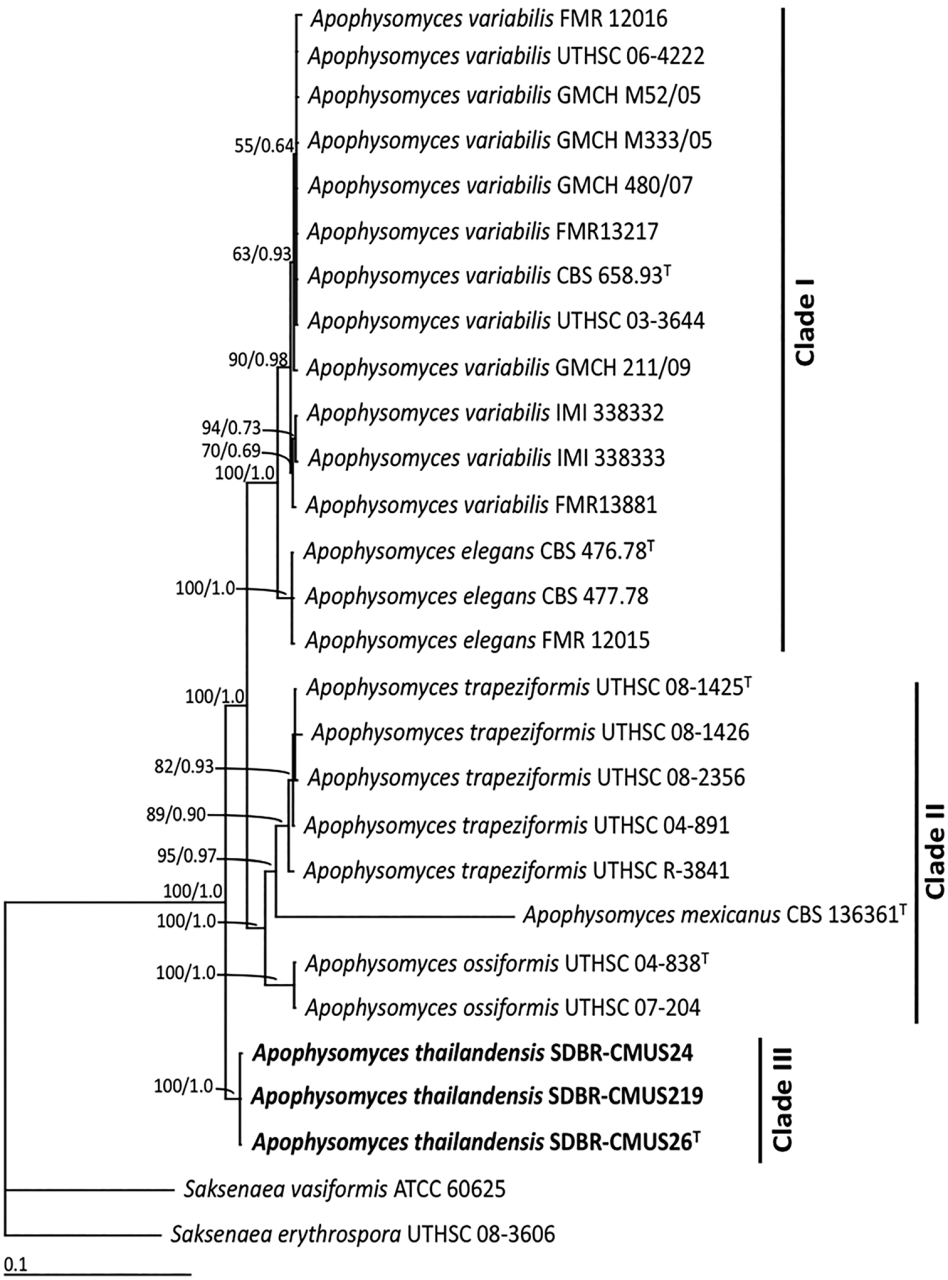
Phylogenetic tree derived from maximum likelihood analysis of a combined ITS, LSU, and H3 genes of 28 sequences. *Saksenaeavasiformis* and *S.erythrospora* were used as outgroup. Numbers above branches are the bootstrap statistics percentages (left) and Bayesian posterior probabilities (right). Branches with bootstrap values ≥ 50% are shown at each branch and the bar represents 0.1 substitutions per nucleotide position. The fungal isolates from this study are in bold. Superscript T = type species.

The topologies of each single-gene and the multi-gene (ITS, LSU, and H3 genes) trees were similar. Therefore, we show only the multi-gene tree (Fig. 1). Our phylogenetic analysis separated *Apophysomyces* into three main clades. Clade I contained two species (*A.variabilis* and *A.elegans*). *Apophysomycestrapeziformis*, *A.mexicanus*, and *A.ossiformis* were assigned to clade II. *Apophysomycesthailandensis* was clearly separated from the other *Apophysomyces* species and formed a separate monophyletic clade (clade III) with high BS (100%) and PP (1.0) support.

The percentage of nucleotide distances of ITS (ITS1+5.8S+ITS2) sequence between *A.thailandensis* and other *Apophysomyces* species is shown in Table 5. The percentage nucleotide distance of *A.thailandensis* ranged from 4.53–15.60% from other *Apophysomyces* species.

**Table 5.** Mean percentage nucleotide *p*-distances of ITS (ITS1+5.8S+ITS2) sequences compared between *Apophysomyces* species.

Number	*Apophysomyces* species	Within species	1	2	3	4	5
1	*A.thailandensis* (n=3)	0.0 ± 0.00					
2	*A.trapeziformis* (n=5)	1.15±0.31	4.53±0.43				
3	*A.ossiformis* (n=2)	0.10±0.00	5.25±0.07	4.70±0.28			
4	*A.variabilis* (n=12)	0.55±0.26	4.96±0.05	5.85±0.24	5.95±0.13		
5	*A.mexicanus* (n=1)	–	15.60±0.00	16.30±0.00	15.30±0.00	16.10±0.00	
6	*A.elegans* (n=3)	0.10±0.00	4.56±0.26	6.18±0.17	5.75±0.21	3.00±0.10	16.75±0.38

### Metal minerals solubilization ability

**Figure 2.**
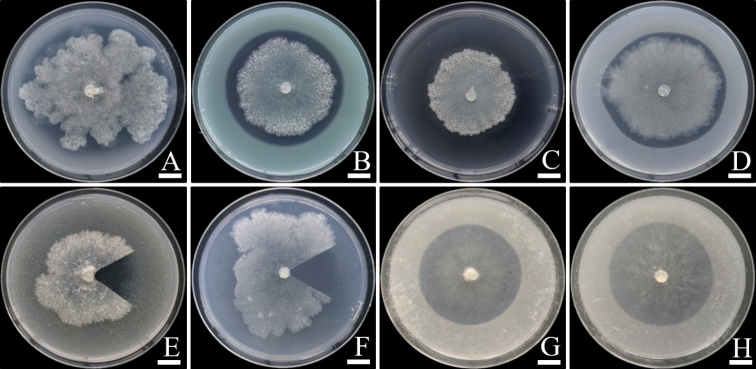
Solubilization of non-soluble minerals in agar media by *Apophysomycesthailandensis* SDBR-CMUS26 (holotype). **A** Ca_3_(PO_4_)_2_**B** CuCO_3_•Cu(OH)_2_**C** CuO **D** ZnCO_3_**E** FePO_4_**F** MnO **G** Feldspar **H** Kaolin. Scale bars: 10 mm. Fungal colonies in **E** and **F** were cut for the solubilization area (halo zone) observation.

**Figure 3.**
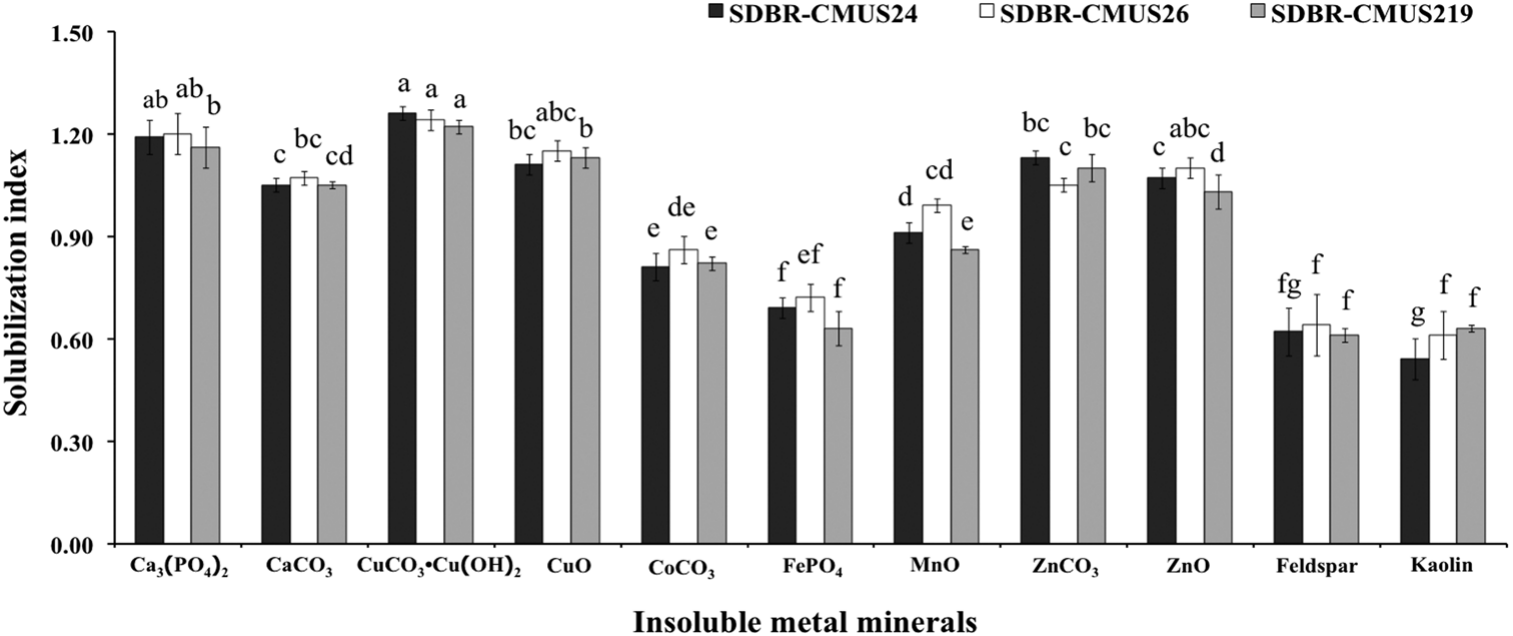
Solubilization index of the ability to solve non-soluble mineral by *Apophysomycesthailandensis*. Data are means of three replicates. Error bar at each point indicates ± SD. Different letters above each graph indicate that the means are significantly different by Tukey’s test (*P* < 0.05)

The ability of *A.thailandensis* to solubilize metal minerals depended on the type of minerals and strain. In some cases, *A.thailandensis* produced a solubilization zone in agar that was larger than the fungal colonies (Fig. 2A–D), while in other cases the solubilization zones were found beneath the fungal colonies (Fig. 2E–H). The solubilization activities were expressed in terms of a solubilization index (SI) and are shown in Figure 3. The solubilization activity of all *A.thailandensis* strains in the presence of CaCO_3_, Ca_3_(PO_4_)_2_, CuCO_3_•Cu(OH)_2_, CuO, ZnCO_3_, and ZnO was characterized as medium (SI value between 1.0 and 2.0) activity. All strains showed a low solubilization activity (SI value less than 1.0) for CoCO_3_, FePO_4_, MnO, feldspar, and kaolin.

### Taxonomy

#### Apophysomyces thailandensis

Taxon classificationFungiMucoralesSaksenaeaceae

S. Khuna, N. Suwannarach & S. Lumyong sp. nov.

827677

Fig. 4

##### Etymology.

For ‘*thailandensis*’, referring to Thailand, where soil containing the new fungus was collected.

##### Holotype.

THAILAND. Chiang Mai Province: Mae Wang District, (18°36'46"N, 98°46'30"E), isolated from soil in agricultural area, 8 August 2017, S. Khuna, dried cultures: SDBR-CMUS26; ex-type living culture: TBRC9299

##### Gene sequences

**(from holotype).** MH733251 (ITS), MH733254 (LSU), MH733257 (H3).

##### Diagnosis.

Distinguished from other *Apophysomyces* species by the slightly trapezoidal sporangiospores, and from *A.elegans*, *A.trapeziformis*, and *A.mexicanus* by its narrower sporangiospores.

**Figure 4.**
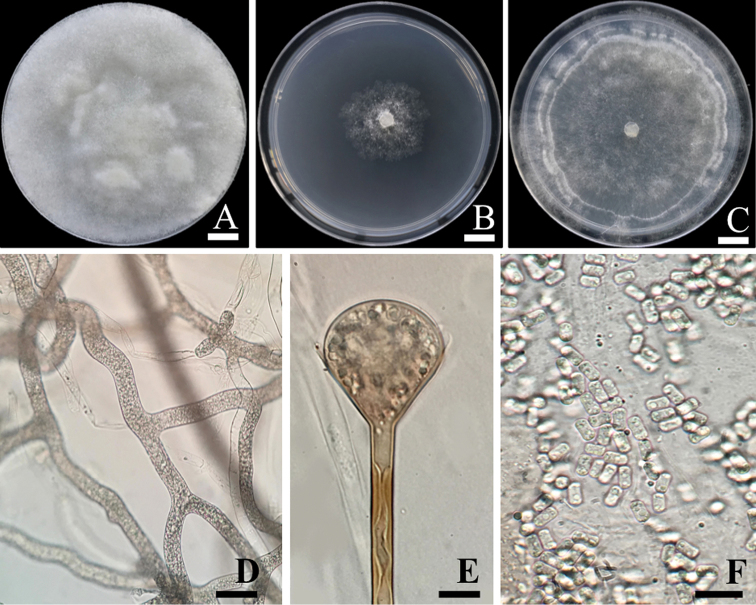
*Apophysomycesthailandensis* SDBR-CMUS26 (holotype). **A** colony on potato dextrose agar **B** Colony on malt extract agar **C** Colony on Czapek agar **D** Branched, aseptate hyphae **E** Funnel-shaped apophysis **F** Slightly trapezoidal sporangiospores. Scale bars: 10 mm (**A–C**), 10 µm (**D–E**), 20 µm (**F**).

Colonies on PDA attaining a diameter of 90 mm after 2 d at 37 °C, whitish at first, becoming white to cream-colored, reverse concolorous (Fig. 4A). Colonies on MEA attaining a diameter of 90 mm after 5 d at 37 °C, flat, whitish, reverse concolorous (Fig. 4B). Colonies on CZA attaining a diameter of 90 mm after 4 d at 37 °C, whitish at first, becoming white to cream-colored, with scarce aerial mycelium, reverse concolorous (Fig. 4C). On all agar media the hyphae are branched, hyaline, smooth-walled, and have 5–15 µm in diameter (Fig. 4D). Sporulation was observed only on CZA. Sporangiophores erect, usually arising singly, emerging from aerial hyphae, at first hyaline but soon becoming light brown, usually straight, slightly tapered towards the apex, unbranched, 60–890 µm in length, 3.75–7.5 µm wide, and smooth-walled. Sporangia apophysate, terminal, pyriform, multispored, white at first, becoming light greyish brown when mature, and 25–58 µm in diameter. Apophyses short, funnel to bell shaped, 21–52 × 19–46 µm (Fig. 4E). Sporangiospores slightly trapezoidal in side view, cylindrical in front view, with flattened to slightly concave lateral walls, hyaline to light brown in mass, smooth- and thin-walled, 5–6(9) × 2–3 µm (Fig. 4F).

##### Other cultures examined.

THAILAND. Chiang Mai Province: Mae Wang District, (18°36'46"N, 98°46'30"E), isolated from soil in agricultural areas, 8 August 2017, S. Khuna, living cultures: SDBR-CMUS24 and SDBR-CMUS219.

### Key to *Apophysomyces* species^1^

**Table:** 

1	Sporangiospores trapezoid, ellipsoid, subtriangular or claviform in shape	*** A. variabilis ***
–	Sporangiospores less variable in shape	**2**
2	Sporangiospores slightly trapezoidal to trapezoidal in shape	**3**
–	Sporangiospores other shapes	**5**
3	Sporangiospores 2–3 µm wide	*** A. thailandensis ***
–	Sporangiospores 3–5 µm wide	**4**
4	Apophyses cup-funnel shape, 8–15 µm long	*** A. mexicanus ***
–	Apophyses funnel-shaped, 15–20 µm long	*** A. trapeziformis ***
5	Sporangiospores bone-like in shape	*** A. ossiformis ***
–	Sporangiospores ovoid, broadly ellipsoidal to barrel-shaped	*** A. elegans ***


## Discussion

The present study identifies a new species of *Apophysomyces*, a soil fungus from Thailand based on morphological and physiological characteristics as well as on phylogenetic analyses. *Apophysomycesthailandensis* is characterized by its funnel- to bell-shaped apophyses and slightly trapezoidal sporangiospores. These morphological characteristics support its placement into the genus *Apophysomyces* (Misra et al. 1979; Alvarez et al. 2010; Bonifaz et al. 2014). Based on morphology, the slightly trapezoidal sporangiospores of *A.thailandensis* clearly distinguish it from *A.elegans* and *A.ossiformis*, with exceptions of *A.mexicanus*, *A.trapeziformis*, and *A.variabilis* (Table 6). However, the width of sporangiospores of *A.thailandensis* (2–3 µm wide) was found to be narrower than *A.elegans* (3–8 µm wide) (Misra et al. 1979; Alvarez et al. 2010), *A.ossiformis* (3–5.5 µm wide) (Alvarez et al. 2010), and *A.variabilis* (3–6 µm wide) (Alvarez et al. 2010).

**Table 6.** Origin, isolation source and microscopic observation of *Apophysomyces* species.

*Apophysomyces* species	Origin	Isolation source	Microscopic observation				
			Hyphae width (µm)	Sporangiophores (µm)	Sporangia (µm)	Apophyses shape / size (µm)	Sporangiospore shape / size (µm)
* A. elegans * ^a, b^	India	Soil	3.4–8	400–540 × 3.4–7.5	20–60	Funnel to bell / 10–46 × 11–46	Ovoid, broadly ellipsoidal to barrel-shaped / 5.4–12 × 3–8
* A. mexicanus * ^c^	Mexico	Human necrotic lesion	3–5.5	100–700 × 3.5–7.0	25–30	Cub-funnel / 12–20 × 8–15	Slightly trapezoidal / 5–10 × 3–4
* A. ossiformis * ^a^	USA	Cellulitis of human leg wound	3–5.5	100–400 × 2–3.5	15–50	Funnel / 15–20 × 15–20	Bone-like / 6–8 × 3–5.5
* A. trapeziformis * ^a^	USA	Abdominal abscess of human	3–5.5	400 × 2–3.5	15–50	Funnel / 15–20 × 15–20	Trapezoid / 5–8.5 × 3–5
* A. thailandensis * ^d^	Thailand	Soil	5–15	60–890 × 3.75–7.5	25–58	Funnel to bell / 21–52 × 19–46	Slightly trapezoidal / 5–9 × 2–3
* A. variabilis * ^a^	Netherlands	Osteomyelitis of human	3–5.5	100–400 × 2–3.5	15–50	Funnel / 15–20 × 15–20	Trapezoid, ellipsoid, subtriangular or claviform / 5–14 × 3–6

^a^Alvarez et al. 2010, ^b^Misra et al. (1979), ^c^Bonifaz et al. (2014) and ^d^This study.

Carbon assimilation profiles have been shown to be useful for differentiation of mucoralean genera (Schwarz et al. 2007). The current study found that *A.thailandensis* showed negative results for D-galactose, amygdalin, arbutin, salacin, and gentiobiose assimilation. This agrees with a previous study, which reported that the genus *Apophysomyces* could not assimilate these five substances (Schwarz et al. 2007; Alvarez et al. 2010; Bonifaz et al. 2014) (Table 4). *Apophysomycesthailandensis* was positive in the assimilation of D-adonitol, D-arabitol, D-fructose, D-glucose, D-mannitol, D-mannose, D-maltose, D-ribose, D-sorbitol, D-trehalose, D-xylose, glycerol, glycogen, *N*-acetyl-glucosamine and xylitol, similar to other *Apophysomyces* species (Alvarez et al. 2010; Bonifaz et al. 2014). However, the assimilation of D-fucose, D-tagatose, D-turanose, and L-fucose and the non-assimilation of L-arabitol by *A.thailandensis* differs from the other *Apophysomyces* species (Alvarez et al. 2010; Bonifaz et al. 2014) (Table 4). The positive results in the nitrogen assimilation profiles and tolerance to various chemical agents for arginine, creatine, L-cysteine, L-leucine, L-lysine, L-ornithine, L-proline, L-tryptophan, nitrate, and 2% MgCl_2_ of *A.thailandensis* are similar to other *Apophysomyces* species (Table 4) (Alvarez et al. 2010; Bonifaz et al. 2014). Nitrite assimilation and 2% NaCl intolerance of *A.thailandensis* separated it from the other *Apophysomyces* species (Alvarez et al. 2010; Bonifaz et al. 2014).

In the phylogenetic analysis based on multi-gene sequences of combined ITS, LSU, and the histone 3 gene, *A.thailandensis* formed a monophyletic clade, separate from the other *Apophysomyces* species. The ITS (ITS1+5.8S+ITS2) genetic distance between *A.thailandensis* and other *Apophysomyces* species ranged from 4.53% to 15.60% (Table 5). This genetic distance of ITS was greater than 3%, which is sufficient to indicate a new fungal species (Leavitt et al. 2013; Nilsson et al. 2008).

In the terrestrial environment, fungi play important roles in the biogeochemical cycling of elements (Gadd 2017; Frąc et al. 2018). Soil fungi can mobilize and solubilize non-soluble minerals into forms available for cellular uptake and leaching from the system, e.g. complexation with organic acid, other metabolites and siderophores (Gadd 2010; Mapelli et al. 2012). In this study, pure cultures of *A.thailandensis* were able to solubilize different non-soluble minerals (Ca, Co, Cu, Fe, Mn, and Zn-containing minerals), and the solubilization demonstrated very different activities for the different minerals. This is similar to previous studies that reported other mucoralean genera (e.g. *Absidia*, *Cunninghamella*, *Mucor*, and *Rhizopus*) isolated from soils are able to solubilize non-soluble minerals (Ca, Fe, Mg and Zn-containing minerals) (Arrieta and Grez 1971; Kolo and Claeys 2005; Akintokun et al. 2007; Nenwani et al. 2010; Sharma et al. 2013; Patel et al. 2015; Alori et al. 2017; Ceci et al. 2018). This is the first report describing non-soluble mineral solubilization ability by the genus *Apophysomyces*.

In conclusion, the combination of morphological and physiological characteristics, and the molecular analysis strongly support our claim of a new fungus species. This discovery is considered important in terms of stimulating the investigations of soil fungi in Thailand and will help researchers to better understand the distribution and ecology of the genus *Apophysomyces*.

## Supplementary Material

XML Treatment for Apophysomyces thailandensis

## Footnotes

Sporangiospores and apophyses observed on Czapek agar in culture.
